# MicroRNA-34a and vascular senescence in diabetes

**DOI:** 10.18632/aging.102625

**Published:** 2019-12-22

**Authors:** Menaka C. Thounaojam, Manuela Bartoli

**Affiliations:** 1 Department of Ophthalmology, Medical College of Georgia, Augusta University, Augusta, GA 30912, USA

**Keywords:** miR-34a, vasculature, senescence, diabetes

Diabetes is a prominent chronic condition characterized by increased glycemic levels due to either loss of insulin production (type I diabetes, T1D) or impaired insulin sensitivity (type II diabetes, T2D). A major target of diabetes is the vasculature and virtually all diabetic complications are associated with macro- and micro-vascular alterations. Therefore, the deleterious impact of hyperglycemia on the vascular endothelium represents the main cause of morbidity and mortality in diabetes.

Recent work has emphasized that diabetes stimulates vascular changes similar to those observed during aging [1]. This was particularly evidenced in experimental models where diabetes/hyperglycemia was imposed in young animals leading to up-regulation of a number of senescence-like features, particularly in microvascular beds [2]. Accelerated vascular aging in diabetes appears to involve a complex sequence of molecular events known as stress-induced premature senescence (SIPS) and culminating in the cellular acquisition of the so-called senescence-associated secretory phenotype (SASP) [2]. This is an irreversible and deleterious process where endothelial cells (ECs) acquire senescence-like features, including up-regulation of cyclin-dependent kinase inhibitors (i.e. p21^Waf1^ and p16^Ink4a^), increased production of cytokines and chemokines and altered expression of degradative enzymes (i.e. matrix metalloproteinase) and of extracellular matrix proteins [3]. Acquisition of SASP in ECs will affect cell survival and, most importantly, is likely to influence the surrounding environment by paracrine activity of the senescence-associated pro-inflammatory secretome [4].

As seen in physiological aging, diabetes-induced SIPS in endothelial cells is thought to be secondary to oxidative stress [2] and is linked to decreased expression and activity of the NAD^+^-dependent histone deacetylase sirtuin-1 (silent mating type information regulation 2 homolog) (SIRT1) [2]. The latter is a key regulator of mitochondrial function and biogenesis [5], as such, loss of SIRT1 is believed to play a critical role in the induction of SIPS [6].

SIRT1 expression has been shown to be regulated by epigenetic mechanisms involving microRNAs (miRs). We have recently shown that microRNA-34a (miR-34a) is up-regulated in the diabetic retina [2] and its overexpression in human retinal endothelial cells (huREC) is associated with the induction of SIPS [7]. Although initially identified in cancer, miR-34a has been implicated in aging including vascular senescence and evidence is provided showing that SIRT1 is a direct gene target of miR-34a [6,7]. Of interest, in studies conducted in senescent retinal pigmented epithelial cells, miR-34a was shown to promote oxidative stress in these cells by altering the SIRT1/p66shc pathway [8].

Our recent work in glucidic stress-induced retinal microvascular ECs senescence demonstrated that elevation of miR-34a in the diabetic milieu results in down-regulation of mitochondrial function and biogenesis due to direct targeting of SIRT1 gene by this miR [7]. Most importantly, we have found that in the glucidic milieu miR-34a suppresses the endogenous antioxidant capacity of retinal endothelial cells by downregulating the expression of the mitochondrial enzymes thioredoxin reductase 2 (TrxR2) and superoxide dismutase 2 (SOD2). 3’-UTR reporter assays confirmed that in huREC only TrxR2 is a direct target of miR-34a, whereas loss of SOD2 appears to be a consequence of miR-34a-mediated suppression of SIRT1 gene expression.

In conclusion, our data and those of others strongly support the notion that miR-34a acts at multiple levels to ultimately enhance endothelial cells susceptibility to oxidative damage, thus, representing a key link between metabolic stress/diabetes and accelerated vascular senescence.
